# Information Needs and Visitors' Experience of an Internet Expert Forum on Infertility

**DOI:** 10.2196/jmir.7.2.e20

**Published:** 2005-06-30

**Authors:** Wolfgang Himmel, Juliane Meyer, Michael M Kochen, Hans Wilhelm Michelmann

**Affiliations:** ^2^Department of Obstetrics and Gynaecology (Study Group of Reproductive Medicine)University of GoettingenGoettingenGermany; ^1^Department of General PracticeUniversity of GoettingenGoettingenGermany

**Keywords:** Consumer health informatics, telemedicine, Internet, e-health, infertility, remote consultation

## Abstract

**Background:**

Patients increasingly use health portals and Web-based expert forums (ask-the-doctor services), but little is known about the specific needs of Internet users visiting such websites, the nature of their requests, or how satisfied they are with Internet health experts.

**Objective:**

The aim of this study was to analyze the information requests of (mostly female) patients visiting an Internet expert forum on involuntary childlessness and their satisfaction with the experts' feedback.

**Methods:**

We posted an electronic questionnaire on a website hosting an expert forum on involuntary childlessness. The questionnaire was “activated” whenever a visitor sent a question or request to the expert forum. The survey focused on the reasons for visiting the expert forum and whether the visitors were satisfied with the experts' answers to previously posted questions. The free-text questions of visitors who answered the survey were analyzed using Atlas-ti, a software program for qualitative data analysis.

**Results:**

Over a period of 6 months, 513 out of 610 visitors (84%) answered the questionnaire. The majority of respondents (65.5%) expected general information about involuntary childlessness, conception, or an evaluation of drugs. Others were concerned about their actual treatment (40.6%) and therapeutic options (28.8%). Out of 225 respondents who had previously contacted the forum, 223 had received an answer, and 123 (55.2%) were satisfied with the experts' answers. About half (105/223) of those users who had previously received an answer from the expert forum stated that they had discussed it with their own doctor. More of these users were satisfied with their subsequent care in fertility clinics than users who did not talk to their doctor about their Internet activities (93.9% vs 76.1%; *P* = .015 ). According to the qualitative analysis, many requests (n = 194) were more or less trivial, especially those for information on basic aspects of reproduction. More than one-third of visitors (n = 199) sent detailed results of diagnostic tests and asked for a first or second opinion. Requests to the expert forum were also sent in order to obtain emotional support (17%) or to complain about a doctor (15%).

**Conclusions:**

Visitors who sent their laboratory findings to receive a thorough evaluation or a second opinion had a good command of the opportunities that an expert forum offers. One important expectation of the forum was emotional support, indicating psychological needs that were not met by medical providers. Future websites must find a compromise in order to protect experts from being overwhelmed by general, nonspecific requests while supporting patients with individualized answers.

## Introduction

Both healthy and sick people increasingly use electronic media to get medical information and advice [1]. One out of four Europeans search the Internet to receive information about health, and more than 40% consider this a reliable way to obtain information [2]. In a survey of nearly 5000 Internet users drawn from the US Research Household Panel, 40% used the Internet for advice or information about health, and 6% used it to communicate with health professionals [3].

The Internet has the potential to help patients to become active and well informed, instead of being passive health care consumers [4,5]. Van Woerkum [6] considers this in terms of a sender-receiver model: the Internet user is not only a receiver, but is active in solving a problem via the Internet. The user actively exchanges information with others about a subject of interest.

There are several reports about why consumers visit certain websites or expert forums. In their analysis of electronic mail sent to the webmaster of a cardiac website, Widman and Tong [7] found that most inquiries were about therapy and diagnosis, and only a few were about patient education. A content analysis of unsolicited electronic mail sent to a dermatological website concluded that emails contained questions about a particular treatment (30%), new therapies (12%), or about specialists for the treatment of a specific disease (15%). Most inquiries pertained to general information about a specific disease (34%) [8]. It appears that many visitors seem to consult a website looking for a second opinion [7,8].

According to a recent study on the Swedish public health service Infomedica [9,10], most people consulted the Internet expert forum “Ask the Doctor” to receive a second opinion (31%), especially because they were unsatisfied with their doctor (25%). Few (15%) consulted the forum for a primary evaluation of a medical problem. Accessing this service at their own convenience was the feature most appreciated by visitors (52%). Based on a qualitative content analysis of visitors' questions, information and advice were the most frequent reasons to visit a University of Washington health education website offering information about orthopedics and sports medicine [11].

Even with all this research, we still know very little about the specific needs of Internet users visiting medical websites, the nature of their requests, or how satisfied they are with the Internet service. Therefore, it is difficult to form valid conclusions about consumer health informatics or electronic communication services and their impact on personal health, patient information, and the clinician-patient relationship. More detailed information, derived from qualitative and quantitative methods, could help to reveal health needs not covered by traditional outpatient or hospital services [12].

In this study we used a qualitative approach to analyze in detail the needs and expectations of patients visiting a specialized health website. Furthermore, we investigated the visitors' experience and satisfaction with the offered service using a quantitative method. For these analyses, we chose a website about involuntary childlessness for two reasons:

1. The burden of involuntary childlessness is high [13] and so is the number of patients using the Internet as an outlet for talking about infertility [14].
2. There are numerous therapeutic options for treating infertility; therefore, patients are often confronted with the question of which treatment might be most successful in their specific situation [15].

## Methods

The study design comprised two phases:

- a Web-based survey of visitors who sent a request to the Internet expert forum, and
- a content analysis of these requests.

### Setting

The study was conducted on the German website www.rund-ums-baby.de, which provides information for parents and potential parents. The site consists of several sections, such as reproduction, pregnancy, birth, and parenting. In each section, visitors can refer to a group of medical experts (expert forum) and ask questions directly via a Web-based interface or by email. In the section “Wish for a Child,” the expert team consists of six to eight experts who are board certified in gynecology, urology, andrology, or embryology. Some of them work in an outpatient department, some in reproductive clinics, and some in university hospitals. The experts' work with the forum is on a voluntary, unpaid basis. It is possible for visitors to find the experts' addresses on the website but, to our knowledge, it is unusual for them to personally visit an expert in his or her surgery or clinic. Until 2003 (including the study period), visitors did not have to pay to ask a question. At the time of writing, a nominal fee of 2 euros is charged.

If visitors send a request to one of the experts, the request (without any email address) and the answer are openly published on the website. Further comments from any visitor to the site are welcome and are also published. The structure of these dialogues resembles, for example, The Heart Forum of the Cleveland Clinic Foundation [16]. A PowerPoint presentation about the website can be found in Multimedia Appendix 1. There are several other online sources for infertility-related problems in Germany, but until 2003, www.rund-ums-baby.de was the only one combining general information and expert advice.

To date, more than 10000 electronic messages have been published on the website. Of these, 3840 could be identified as original requests to the expert forum (excluding expert answers, comments, demands, or requests that had nothing to do with involuntary childlessness). The first names of the visitors indicated that only 69 requests (1.8%) were from men.

### Open Survey

We posted a questionnaire on the website from August 27, 2001 to February 28, 2002. The questionnaire was “activated” whenever a visitor sent a request to the expert forum. Right at the beginning of the questionnaire, visitors were informed that they were not obliged to answer the questionnaire (informed consent) and were told how they could exit the questionnaire. The questionnaire was designed for adaptive questioning. The request of the visitor and his or her answers to the questionnaire were immediately separated from each other so that the expert team did not know whether a visitor had answered the questionnaire or what the answers were.

The questionnaire comprised 22 items. First, visitors were asked whether they had filled out a questionnaire in the past. Then, in the first set of questions, participants were asked to explain their reasons for visiting the website and the expert forum, whether they had previously sent a request and, if so, how they had used the information and how satisfied they were with the experts' answers. The second set of questions related to the actual treatment situation of the participant. At the end of the questionnaire, participants were asked for some sociodemographic details.

The questionnaire was pilot tested with 30 visitors to the website. They were asked at the end of the questionnaire whether they had any difficulties in answering the questions and whether they had any technical problems handling the questionnaire. None of the respondents reported any problems. The final version of the questionnaire is available in Multimedia Appendix 2 and 3. A non-edited English translation is also provided.

Descriptive statistics were applied to analyze the survey data, including absolute and relative frequencies and cross-tabulations, using SAS 8.2 [17]. Differences between nominal variables were tested for statistical significance using the Pearson chi-square test, with alpha set at *P* < .05.

### Analysis of Requests

Requests of those visitors who had answered the survey were analyzed using Atlas-ti [18], a software program for qualitative data analysis. Single phrases or the whole request were coded according to a list of categories and subcodes that we had developed in a retrospective analysis of former requests to the expert forum. These categories were developed and refined by a multidisciplinary group, consisting of two physicians, an expert in reproductive medicine, and a sociologist (JM, MMK, HWM, WH). In detail, HWM suggested a broad spectrum of categories from his work and experience in the expert forum, which JM transformed into a hierarchy of general expectations of the expert forum and different special requests (“codes”; see Table 4). JM coded the requests according to this list, supervised by WH. To ensure a valid coding process, a list of different examples and their respective codes was produced by JM and adjusted by HWM and WH. Problems in coding were discussed with all authors. Most importantly, we not only coded the “official” request but also implicit messages and expectations regarding the expert forum.

### Data Security

The webmaster for the expert forum was responsible for the handling of the data. He administered all requests and all questionnaires during the study period. Afterwards, the data were securely transmitted via a SSL (secure sockets layer) connection to the Department of General Practice without using any email addresses.

The study was approved by the local ethics committee of the University of Goettingen.

## Results

A total of 513 answers from participants were analyzed. These users had visited the Internet forum, sent a request to one of the experts, and answered the survey.

**Table 1:** Study characteristics compared with the German population*

	**Percent of Study Sample** **Age: 18–43** **(N = 513)**	**Percent of German Population** **Age: 20–45** **(N = 29551600)**
**Sex**		
Female	99.2	48.7
Male	0.8	51.3
**Family Status**		
Married	72.5	49.6
Partnership	26.7	-
Single, divorced, widowed	0.8	50.4
**Education**		
Less than 10 y	12.3	33.6
10 y	40.9	24.6
More than 10 y	19.6	17.3
University degree	27.2	11.3
Other		13.6

^*^ Federal Statistics Office [19]

During the study period, the survey was activated by 1305 visitors, of whom 632 (48.4%) declared that they had already visited the website several times and had previously filled in the questionnaire. Because 53 visitors (4.1%) had no wish for a child, they were excluded from further analysis; 97 visitors refused to participate, giving a response rate of 84.1% (513/610). Nearly all respondents were women. Compared to the German reference population, many more of the respondents lived in stable partnerships and were better educated (Table 1).

### Survey Results

At the beginning of the survey, the visitors were asked how they found the website. About 43% (220/509) found the Internet forum by chance, and 83 visitors had systematically searched the Internet for such a website. Only 6 visitors had received this, or a similar, Internet address from their doctor. More than half of the respondents (276/501) sent a question to the expert forum for the first time, and 225 persons had previously consulted the expert forum.

Most of the respondents who reported suffering from involuntary childlessness had already contacted a gynecologist (361/484). For 15%, however, the expert forum was the first professional contact from which they hoped to receive information. Table 2 presents the respondents' reasons for visiting the expert forum. Most of them asked for general information about involuntary childlessness and conception or had questions about their actual treatment.

Of 225 visitors who had previously contacted the forum, 223 received an expert answer. More than half (55.2%; n = 123) were satisfied with the experts' answers, 7 were unhappy with the reply, and the remainder were undecided. Additional comments about the quality of the expert forum were provided by 65 respondents. Apart from many positive reactions, 31 of these respondents expressed dissatisfaction because they either did not receive a previous answer to their question (n = 13), waited too long for an answer (n = 12), or considered the answers superficial (n = 9), inadequate (n = 5), or difficult to understand (n = 3).

**Table 2:** Self-reported reasons for visiting the Internet expert forum (n = 505)*

**Reason**	**Percent**
General information	72.9
Questions about current treatment	45.1
Questions about different treatment options	32.1
Questions about causes of infertility	25.5
Questions about diagnostic data	22.0
Other	7.7

^*^ Multiple answers possible

About half of the users who received a previous answer from the expert forum (105/223) discussed it with their own doctors, some with their fertility clinic doctor, some with their gynecologist, and some with their general practitioner. Of these users, more of them were satisfied with their subsequent medical treatment and/or consultation than visitors who had not talked to their doctor about their Internet experience. This difference was only significant for patients in fertility clinics (Table 3). A quarter of respondents (51/221) changed their doctor or consulted a specialist because of the experts' answers, and 56 started treatment following the experts' advice.

**Table 3:** Satisfaction with medical provider (% of patients who  said they were satisfied with treatment or consultation)

	**Talked With Doctor About Expert Answer**		
**Medical Provider**	**Satisfied Among Those Who Talked**	**Satisfied Among Those Who Did Not Talk**	*P* value*
Fertility clinic (n = 95)	93.9	76.1	.015
Gynecologist (n = 155)	80.5	74.4	.36
General practitioner (n = 47)	72.4	66.7	.68

^*^ Significance of chi^2^ test

Many respondents to the survey were disappointed that they could not talk with their doctors about psychological problems (n = 79), sexual problems (n = 37), somatic complaints (n = 30), or difficulties in their partnership (n = 30). Those who described complaints about their doctors in detail most often mentioned lack of time during consultation (n = 28) and inadequate information (n = 28). Of the women, 20 were upset about “being treated as a number,” being reduced to their abdomen, or being considered a “laying hen.”

### Content Analysis of Types of Requests

We categorized the requests according to the type of help that visitors sought from the expert forum. Each category had several subcategories (Table 4). Most people sought information about conception, reasons for childlessness, evaluation of drugs, diagnostic procedures, and therapeutic options. Many of these requests were very basic.

110; FB 382.txt With the help of an ovulation calendar I have ascertained my fertile days. But what does that mean? If they are, for example, from Sunday to Thursday should I have intercourse every day from Sunday to Thursday or is it better to do so every second day? Or what should I do to become pregnant as soon as possible? Sorry to ask but I heard totally different things.

Many visitors sent their diagnostic tests results in detail and asked for a second opinion as a check or for decision making.

24; FB 12.txt Our doctor recommends assisted hatching. [A process that may help embryos implant in the uterus during an IVF cycle.] What do you think about this technique? Allegedly it should increase pregnancy rate. I am unsure and afraid of course that if implantation occurs, a malformed child may be the consequence. Do you also use this method?

**Table 4:** Types of requests, according to qualitative analysis (n = 513)*

**Expectation of the Expert Forum**	Categories of Requests*	**n**	**Total**
			
Information and explanation	General information	194	
	More detailed questions	333	
	How to find information	2	
			343
Independent medical advice	Second opinion	199	
	Treatment options	116	
	Diagnostic options	26	
	Cost of treatment	16	
	Other	15	
			226
Compliance authority	Criticizing doctors	76	76
			
Guidance	Requests whether to change a doctor	13	
	Requests whether to consult of doctor	11	
	Recommendation of specialists	10	
	Recommendation of clinics	10	
			36
Emotional support	Expression of feelings	80	
	Looking for new hope	8	
	Looking for fellow sufferer	6	
			90

^*^ Multiple classifications possible

The expert forum was also utilized as a sort of guide to finding an adequate specialist or to getting an answer to the question of whether medical help was necessary at all.

The need for information and the complaints about doctors were often intermingled, giving the expert forum a role of reassurance.

196; FB 565.txt According to a new hormonal analysis, my gynecologist told me that my progesterone values were disastrous. A value of 1000-2800 (???) would be normal, but mine was 47. This is why a pregnancy can be excluded. Unfortunately, those values were not explained to me and no treatment was recommended. Can you please explain this? Maybe I do not ovulate and are my values really so catastrophic? Certainly, I will never consult this doctor again. Do you recommend that I visit a fertility clinic or can I do something myself?

105; FB 377.txt I have the right to know what happened in the operating theatre, or am I wrong?…I only received a copy of the findings from the material which was sent in (from the abrasion). Please explain this to me; I don't understand anything. I have the feeling he kept something back…because the doctor told me, I was as fit as a fiddle…I am even more disturbed, because he refused to give me the surgery report.

Furthermore, the expert forum provided emotional support. Visitors sometimes expressed their feelings by using the words “help” or “cry for help” or other expressions which they wrote in capital letters.

478; FB 1248.txt Treatments: 1. ICSI follicular puncture 09.2000: 14 oocytes, all fertilized, 3 cryopreserved, 2 transferred, NEGATIVE … 5. ICSI follicular puncture; 7 oocytes, 5 fertilized, no cryo!, 3 transferred, NEGATIVE. Maybe this happened because of my endometriosis????. Could you recommend down regulation over a three month period? Maybe an HLA analysis should be done. HELP!!! I don't know how to go on, I am devastated and totally helpless.

Some visitors also hoped to receive help concerning problems in their relationship.

67;FB 294.txt My boyfriend always tells me that nothing will happen if my life is so much dominated by the wish for a child. This always dampens my hopes. He does not understand how I feel. All day long I only think about having a baby.

Compared to the requests from women, the few requests from men differed in only one respect—they were usually much shorter.

## Discussion

Visitors to the Internet site www.rund-ums-baby.de not only required detailed medical advice on specific matters of infertility diagnosis and treatment, but they also asked for general information about reproduction and for second opinions. Furthermore, they considered this forum as a source of emotional support and as a place where they could complain about their current treatment. Though the majority of visitors were satisfied with the experts' work, 44.8% were not fully convinced.

### Limitations

When visitors to the website were presented with the survey, some may have left the site instead of declaring their unwillingness to participate in the study. Others may have claimed that they had already responded to the survey, even though this was their first visit to the website since the survey was offered. Since we did not use cookies or check the IP address to register site visits or to identify potential duplicate entries from the same user—due to the demands of the ethics committee and the highly sensitive issue of involuntary childlessness—it was not possible to calculate exact view rates or participation rates. The reported figure of 84% may overestimate the true response rate.

Because study participants wanted an answer to their requests at the same time as being asked to complete to questionnaire, there may have been some social pressure to respond to the survey. Although we informed the participants that the experts would receive only their requests and not their answers to the survey, some visitors might have evaluated the experts' responses in a more positive light for fear of jeopardizing future requests.

Another source of bias may be that satisfaction with the experts' answers could only be assessed by people who visit the site at least twice; however, those who were highly dissatisfied would have been less likely to visit the site again.

### Study Implications

The most striking result of this study is the broad variety of reasons why visitors contacted the forum and the different types of requests:

1. One group of visitors made full use of the opportunities offered by the expert forum: those who sent their laboratory data to the experts to receive a thorough evaluation or a second opinion. This is in line with other studies on reasons for Internet consultations [8,20]. Many of the visitors explained their condition using medical terms and concepts before asking their question.
2. Many requests were not suited to the expertise of the team of specialists. This was especially true in relation to general information about basic aspects of human reproduction. Obviously, patients contacting this website were not satisfied with the information they received from doctors, partners, parents, school, and the mass media, which resembles findings from an earlier study [8]. As access to the Internet expands, the volume of requests may increase and become a strain on the experts [7].
3. One important expectation of the forum was emotional support, which was the main reason for some requests or which appeared embedded in other requests. Involuntary childlessness often results in stress, anxiety, and insecurity about whether or not to choose an artificial reproductive technology [21]. Epstein et al [14] are sure that expert forums can support infertile people by giving them the chance to communicate their feelings of depression, anxiety, or anger. In contrast, Baur [12] doubts whether email and the Internet are appropriate media for counseling. Therefore, many participants in our study may have adapted to the “technical imperative” of the Internet to exchange or to ask for technical information. Most of them described their request as information seeking. Only our more in-depth analysis made us aware of implicit emotional problems and needs in some of the requests.

The use of the Internet to get medical information and advice reflects a lack of patient information. Patients may not receive adequate information from their doctors because doctors have insufficient time to answer all questions or are unwilling to spend adequate time with the patient [6]. About one-third of respondents in our study were dissatisfied with the information they received from their family doctor or gynecologist, and they complained about their own doctor's professional or emotional incompetence. This was also true in an analysis of emails addressed to a university dermatology hospital, in which 17% of patients expressed frustration with their own doctors [8]. Distrust was also a strong concern in a patient survey in primary care practices in Rhode Island, USA [22]. More than 57% of the patients expressed an interest in using the Internet to find out if their health care provider was giving them the tests and treatments they need, although, to date, only 17.3% reported ever doing this on the Internet. Consequently, 53% of the visitors in our study did not talk with their doctor about the experts' answers. More of these patients were dissatisfied with their further medical treatment compared to those who did talk with their doctors about their Internet activities. However, this association was only significant for patients in fertility clinics, and we should emphasize that visitors who have a good relationship with their doctor may be more likely to both share the answer from the expert forum and rate their subsequent treatment as satisfactory. Further research should clarify whether there is evidence for a causal relationship between the discussion of Internet information with the doctor and satisfaction with further medical treatment.

About half of the visitors were also not fully satisfied with the expert forum. According to Kedar et al [23], a strong motive for using the Internet is the dissatisfaction patients have with the fact that they have to wait too long for treatment to start. Some visitors even reacted disquietingly towards a delay of a few days when waiting for an answer to their requests. Expanding Internet opportunities of this kind may result in even more visitors who are dissatisfied with their doctor's information, and who either get lost in a maze of Internet information or wait for an adequate answer longer than tolerated.

There is some concern that regular use of the Internet is highly correlated with income level and education [24]. This “digital divide” [25] was also evident in our study. One explanation could be that better educated people tend to delay having children and may therefore encounter more infertility problems [26]. It is more likely that this population has more experience in the use of the Internet and is more familiar with writing Internet requests.  As appropriate information is crucial for making health care decisions, especially about new treatment options, Internet-based expert forums may amplify the digital divide.

### Conclusions

Internet-based expert forums are well suited to give medical advice in difficult situations, to provide help in making decisions, and to offer second opinions. There is no legitimate reason why doctors should not support their patients' use of the Internet for this purpose. In addition, doctors should offer their patients an open discussion about all the information they have received.

The Internet seems to be a seismograph for psychological needs that are not met by doctors and which, on the other hand, can hardly be fulfilled by virtual experts. Further research is necessary to find out whether dialogues between visitors in a chat room, for example, would be more supportive in cases of emotional stress [27,28] and would stimulate visitors to take on a more active role by exchanging information with like-minded people.

## Multimedia Appendix 1

Screenshots of www.rund-ums-baby.de

## Multimedia Appendix 2

Questionnaire in German

## Multimedia Appendix 3

Questionnaire (translated into English by the authors, unedited)

## Multimedia Appendix 4

Patient Quotes in German
