# Supplementary figures and images for: Information Needs and Visitors' Experience of an Internet Expert Forum on Infertility
Source: J Med Internet Res. 2005 Jun 30;7(2):e20. doi: 10.2196/jmir.7.2.e20 (PMC1550645; doi:10.2196/jmir.7.2.e20)

## Slide 1
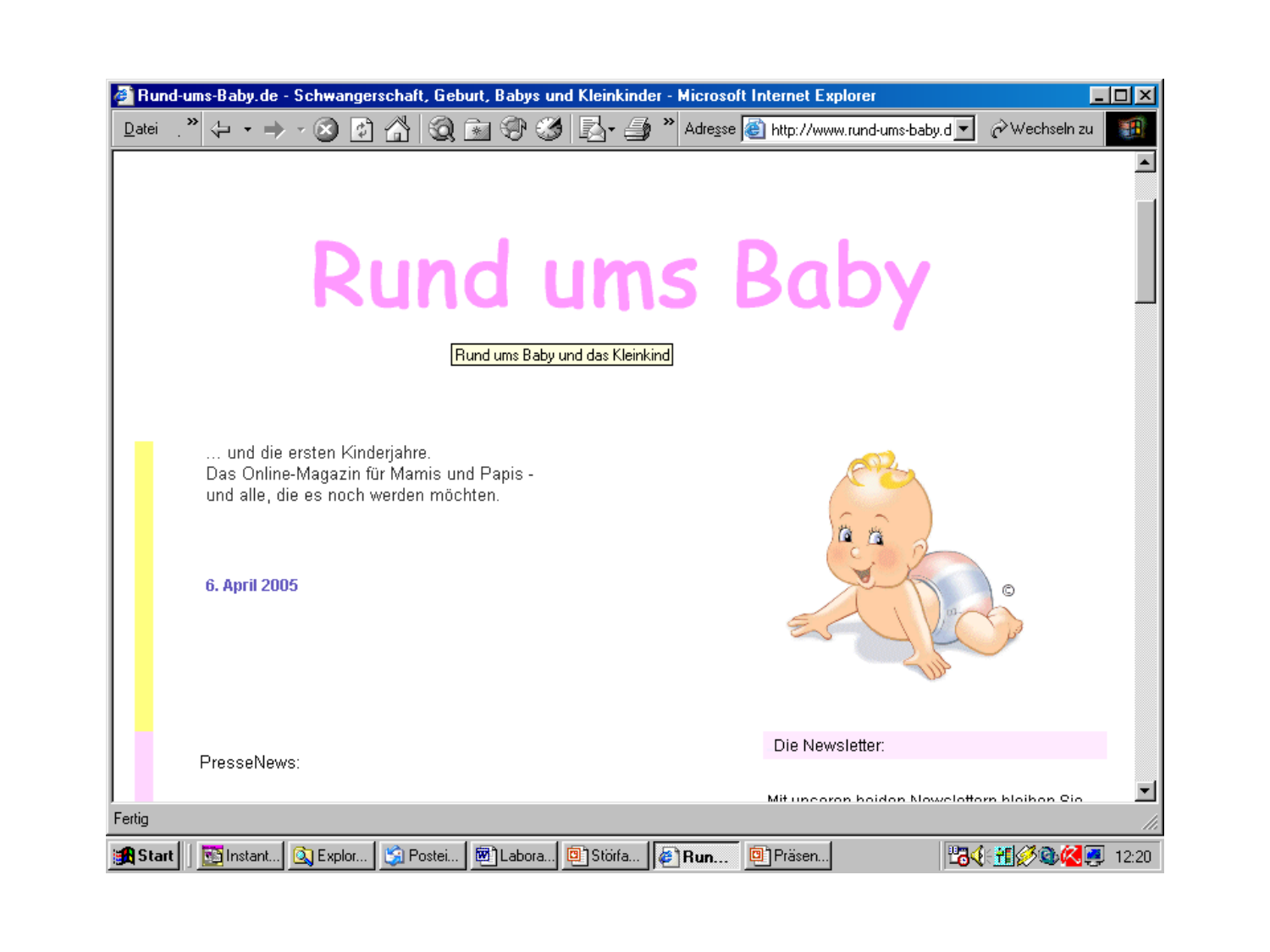

## Slide 2
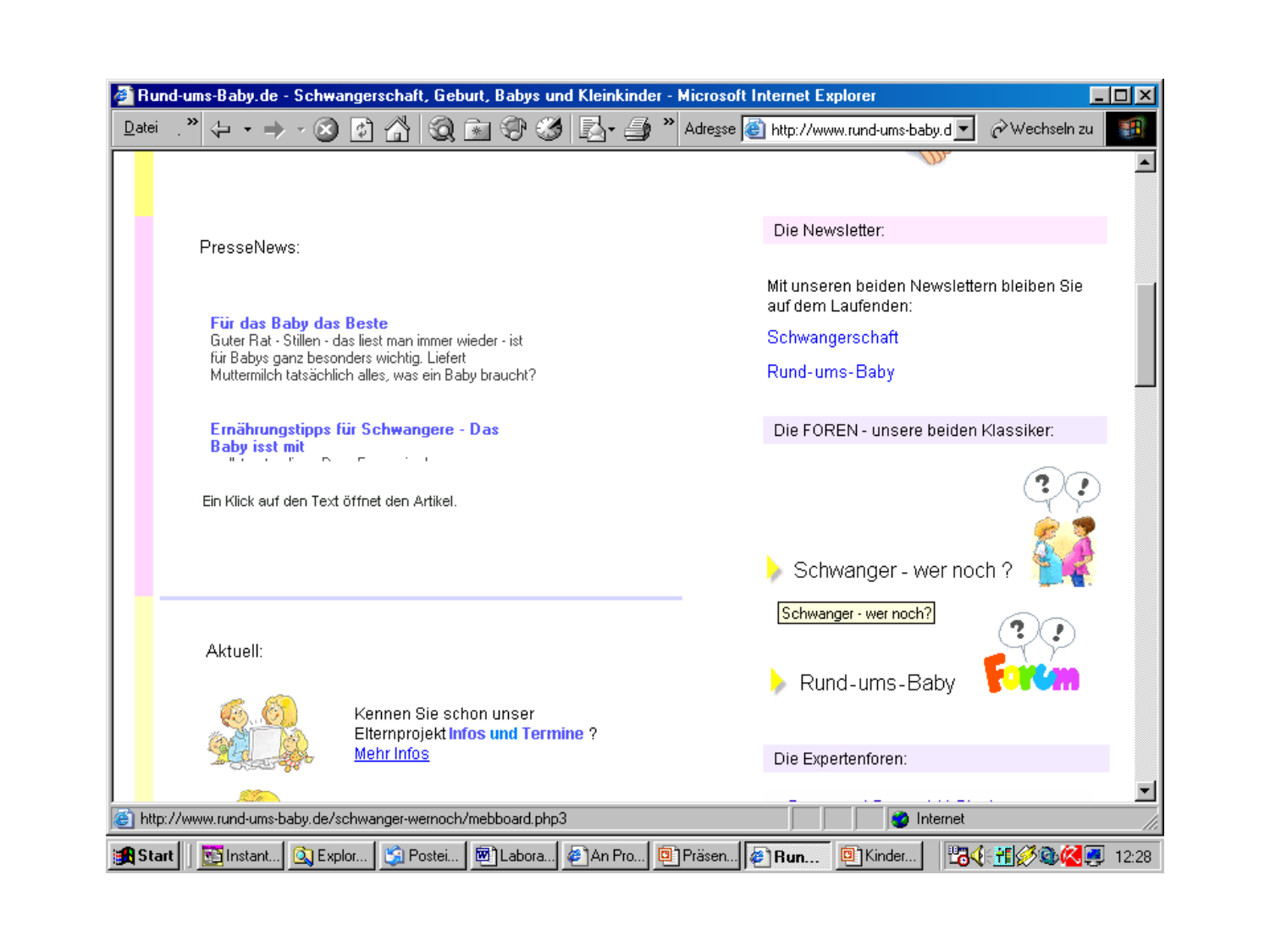

## Slide 3
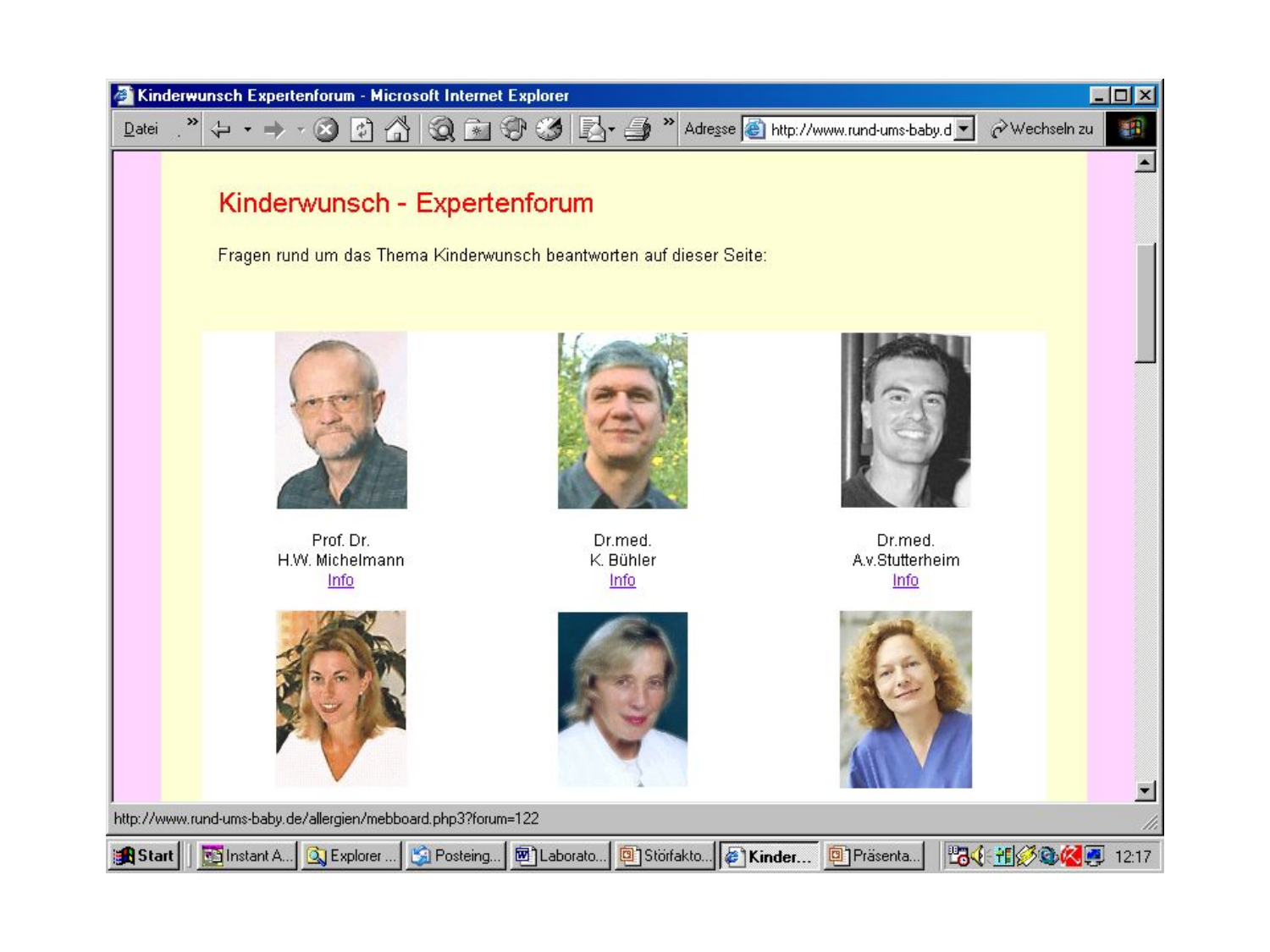

## Slide 4
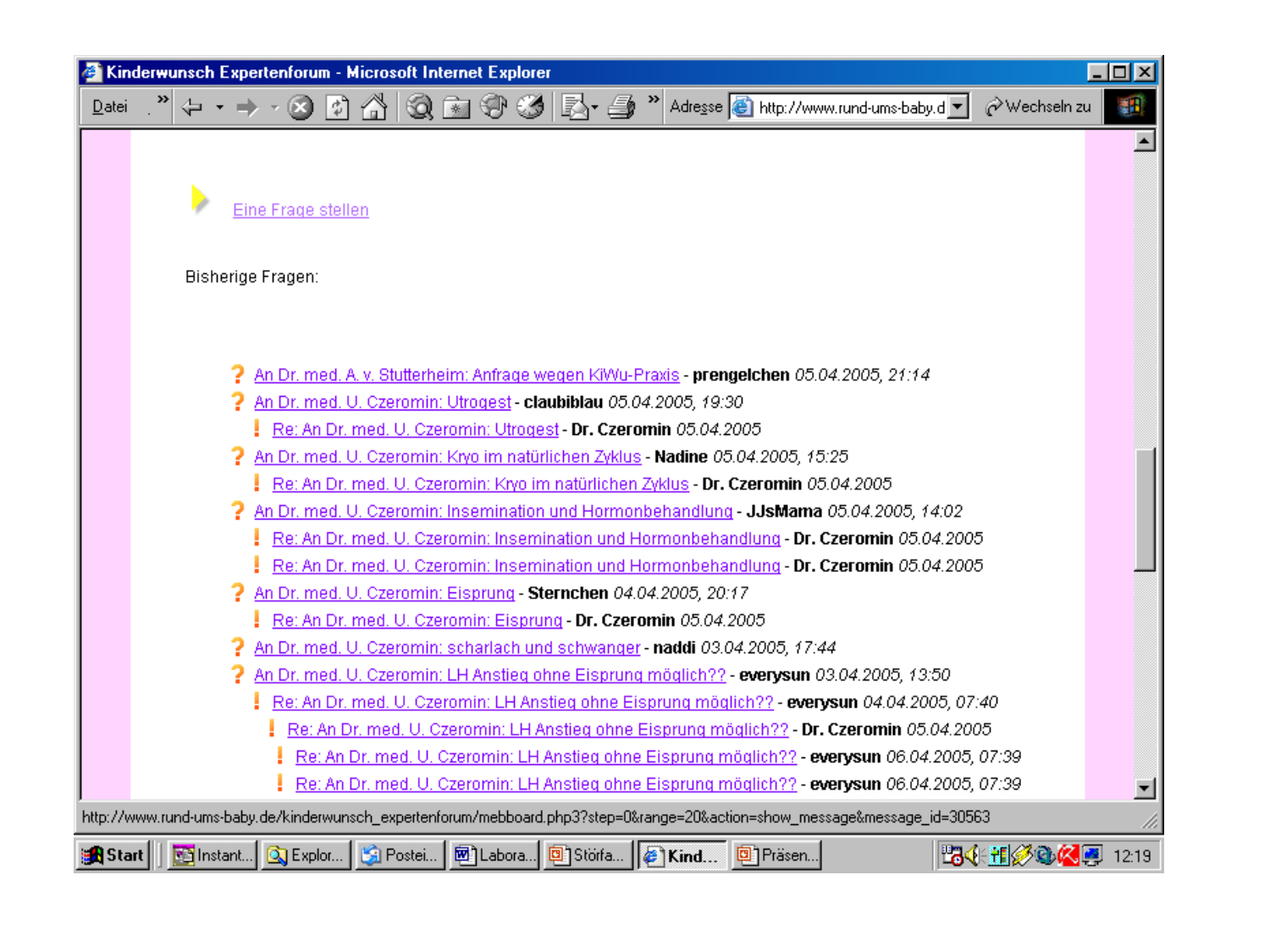

## Slide 5
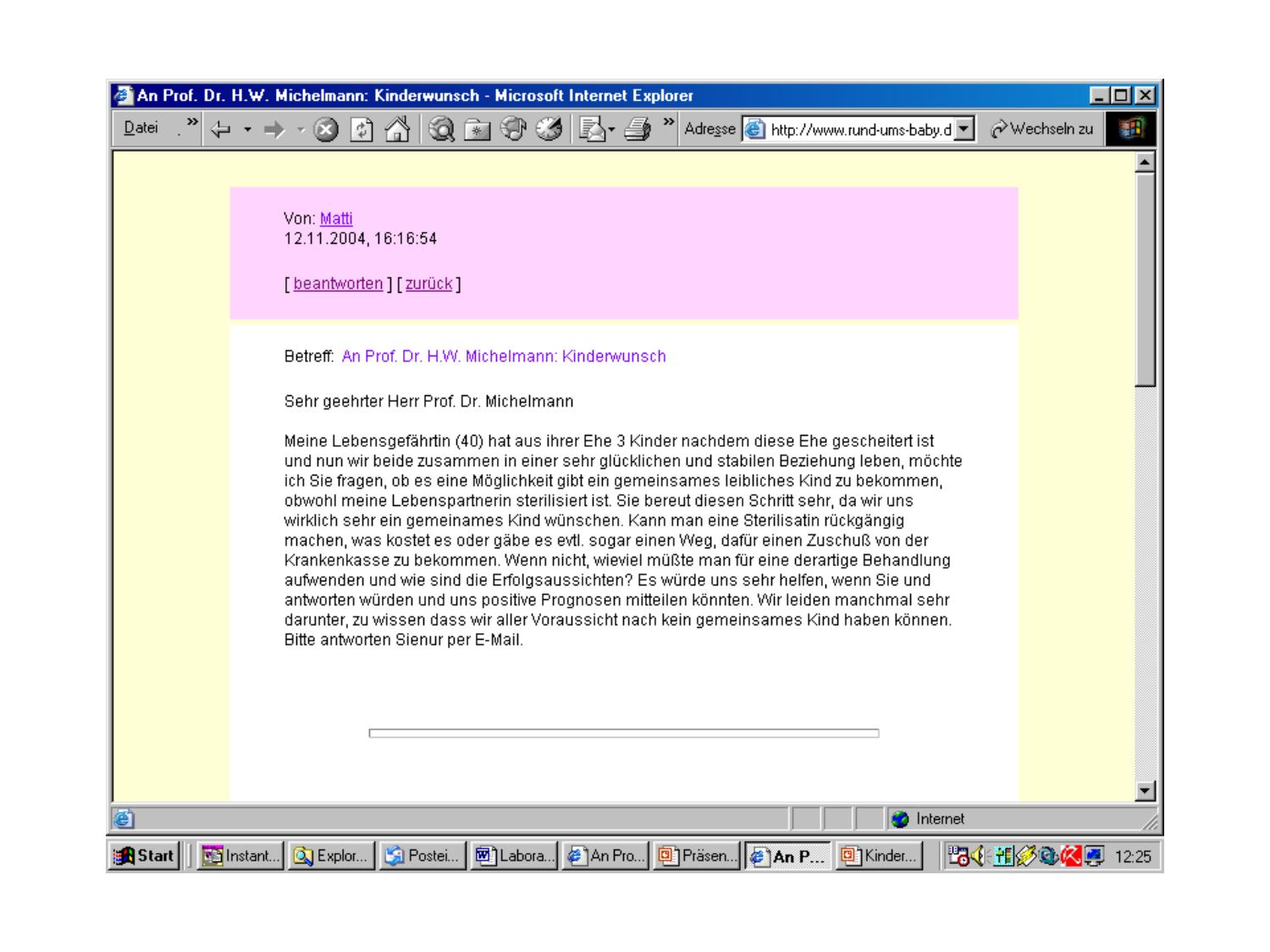

## Slide 6
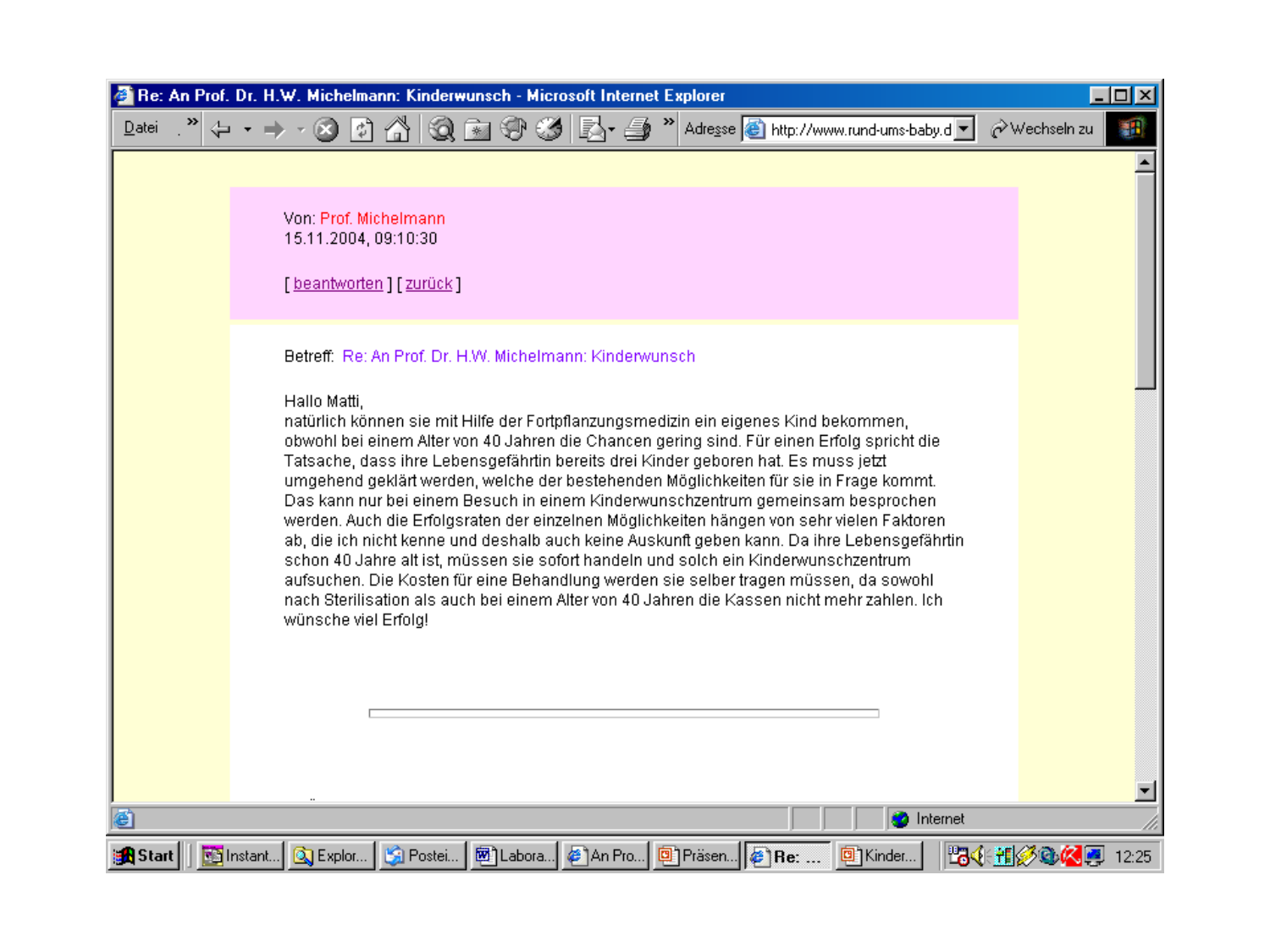

## Slide 7
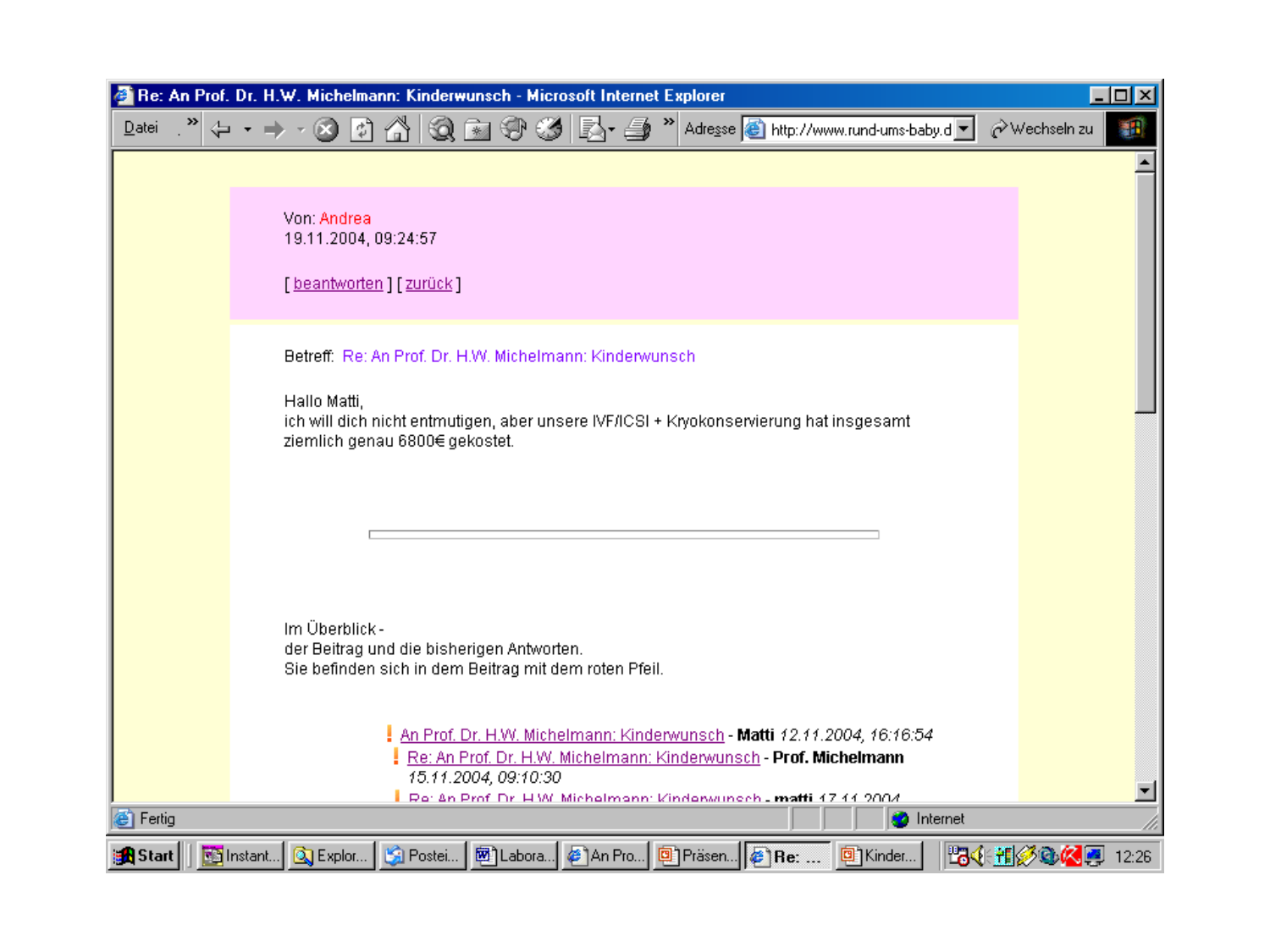

Supplement: Supplementary file 1 [file jmir_v7i2e20_app1.ppt]
