# Supplementary material for: Information Needs and Visitors' Experience of an Internet Expert Forum on Infertility
Source: J Med Internet Res. 2005 Jun 30;7(2):e20. doi: 10.2196/jmir.7.2.e20 (PMC1550645; doi:10.2196/jmir.7.2.e20)
Supplement: Supplementary file 2 [file jmir_v7i2e20_app2.doc]

**Fragebogen**

Liebe Besucherin, lieber Besucher des Expertenforums,

die Abteilung Allgemeinmedizin und die Frauenklinik der Universität Göttingen planen eine Studie über Probleme und Informationswünsche von Frauen und Männern mit unerfülltem Kinderwunsch.

Wir bitten Sie, den nachfolgenden Fragebogen zu beantworten. Ihre Antworten werden von uns streng vertraulich behandelt. Ob Sie den Fragebogen ausfüllen oder nicht: Sie erhalten in jedem Fall eine Antwort auf Ihre Fragen. Aber bitte bedenken Sie, dass Ihnen und anderen Betroffenen die Ergebnisse unserer Untersuchung zugute kommen können.

Wenn Sie mehr über uns wissen oder uns etwas mitteilen möchten, dann finden Sie hier weitere Informationen über die Verantwortlichen dieser Studie [Link] sowie über das Projekt Kinderwunsch [Link] der Abteilung Allgemeinmedizin [Link] und die Fertilitätssprechstunde [Link] der Frauenklinik der Universität Göttingen.

Herzlichen Dank für Ihr Interesse!

[Filterfrage]

*Haben Sie den Fragebogen schon einmal ausgefüllt?*

Ja ⇒ [automatische Rückleitung zum Forum]

Nein ⇒ [automatische Weiterleitung zur nächsten

## Frage]

### Ich habe keinen

unerfüllten Kinderwunsch ⇒ [automatische Rückleitung zum Forum]

*Würden Sie an der Studie teilnehmen und den Fragebogen ausfüllen?*

Ja ⇒ [automatische Weiterleitung zum Fragebogen]

Nein ⇒ [automatische Rückleitung zum Forum]

1. *Wer hat Sie auf dieses Expertenforum aufmerksam gemacht?*

1. Arzt/Ärztin 
2. Krankenschwester/Pfleger/Arzthelferin 
3. Bekannte/Verwandte/Ehemann 
4. Zeitschriften/ Fernsehen 
5. Durch Zufall entdeckt 
6. Systematisch im Netz gesucht 
7. Sonstiges:________________________________________________

2. *Aus welchem Grund besuchen Sie das Expertenforum?*

(Mehrfachnennung möglich)

a) Ich suche Informationen, weil sich mein Kinderwunsch noch nicht erfüllt

hat 

b) Ich habe Fragen zu den Ursachen der Kinderlosigkeit 

c) Ich habe Fragen zu den verschiedenen Behandlungen 

d) Ich habe Fragen zu meiner derzeitigen Behandlung 

e) Ich habe Fragen zu Untersuchungsergebnissen 

f) Sonstiges:__________________________________________________

3. *Stellen Sie heute zum ersten Mal eine Frage an das Expertenforum?*

Ja  [automatische Weiterleitung zu Frage 8]

Nein  [automatische Weiterleitung zu Frage 4]

# 4. *Konnte das Expertenforum Ihre Informationswünsche befriedigen?*

a) Ja 

b) Nein 

c) Teils/teils 

Kritik/Lob und Wünsche an das Expertenforum:

__________________________________________

5. *Besprechen Sie die Antworten der Experten mit Ihrem Arzt/Ärztin?*

**Ja **

**Nein **

6. *Haben die Antworten der Experten Sie veranlasst, zusätzlich einen anderen Arzt/Ärztin oder Spezialklinik aufzusuchen?*

**Ja **

**Nein **

7. *Haben Sie aufgrund des Ratschlags eines Experten eine Behandlung*

*begonnen?*

**Ja **

**Nein **

8. *Wie lange beschäftigt Sie schon das Problem „Kinderlosigkeit“?*

**ca. __ Monate**

**ca. __ Jahre**

9. *Wo suchten Sie* ***zuerst*** *Rat über Ihre Kinderlosigkeit?*

a) Bei diesem Expertenforum 

b) Hausarzt/ärztin 

**c) Frauenarzt/ärztin **

**d) Urologen/in **

e) Bekannte/Verwandte 

f) Geistlichen, Seelsorger 

g) Psychotherapeut/in 

h) Heilpraktiker/in 

i) Sonstige_____________________________________________

10. *Sind Sie in Behandlung?*

Ja  *Wenn Ja, wie lange*: a) seit __Monaten

b) seit __Jahren

Nein  [automatische Weiterleitung zu Frage 16]

11. *Wie werden Sie zur Zeit behandelt? (Mehrfachnennungen möglich)*

a) Hormone 

b) Künstliche Befruchtung (Beispiel IVF, ICSI, IUI) 

c) Psychotherapie 

d) Alternative Methoden (z.B. Akupunktur, Homöopathie) 

e) Operation 

1. f) Beratung 

g) Sonstiges____________________________________________

12. *Wie ist die* ***Betreuung*** *durch Ihren Arzt/Ärztin oder Therapeuten/in während Ihre Behandlung? (Bewerten Sie bitte die Ärzte/Therapeuten, mit denen Sie in Kontakt stehen.)*

##### **Betreuung**

Sehr gut Gut Eher schlecht Sehr schlecht

a) Frauenarzt/ärztin ⭘ ⭘ ⭘ ⭘
b) Hausarzt/ärztin ⭘ ⭘ ⭘ ⭘
c) Arzt/Ärztin in einer

Kinderwunschklinik ⭘ ⭘ ⭘ ⭘
d) Psychotherapeut/in ⭘ ⭘ ⭘ ⭘
e) Androloge/in ⭘ ⭘ ⭘ ⭘
f) Heilpraktiker/in ⭘ ⭘ ⭘ ⭘
g) Sonstige___________ ⭘ ⭘ ⭘ ⭘

13. *Wie fühlen Sie sich durch Ihren Arzt/Ärztin oder Therapeuten/in* ***informiert*** *und* ***aufgeklärt****? (Bewerten Sie bitte die Ärzte/Therapeuten, mit denen Sie in Kontakt stehen.)*

**Informationen/ Aufklärung**

Sehr gut Gut Eher schlecht Sehr schlecht

a) Frauenarzt/ärztin ⭘ ⭘ ⭘ ⭘
b) Hausarzt/ärztin ⭘ ⭘ ⭘ ⭘
c) Arzt/Ärztin in einer

Kinderwunschklinik ⭘ ⭘ ⭘ ⭘
d) Psychotherapeut/in ⭘ ⭘ ⭘ ⭘
e) Androloge/in ⭘ ⭘ ⭘ ⭘
f) Heilpraktiker/in ⭘ ⭘ ⭘ ⭘
g) Sonstige___________ ⭘ ⭘ ⭘ ⭘

###### Ihre Wünsche an den Arzt:______________________________________

14. *Können Sie mit Ihrem behandelnden Arzt/Ärztin oder Therapeuten/in über alle* ***Probleme reden****, die Sie bei der Behandlung haben? (Bewerten Sie bitte die Ärzte/Therapeuten, mit denen Sie in Kontakt stehen.)*

**Ja Zumeist Eher selten Nein**

a) Frauenarzt/ärztin ⭘ ⭘ ⭘ ⭘
b) Hausarzt/ärztin ⭘ ⭘ ⭘ ⭘
c) Arzt/Ärztin in einer

Kinderwunschklinik ⭘ ⭘ ⭘ ⭘
d) Psychotherapeut/in ⭘ ⭘ ⭘ ⭘
e) Androloge/in ⭘ ⭘ ⭘ ⭘
f) Heilpraktiker/in ⭘ ⭘ ⭘ ⭘
g) Sonstige___________ ⭘ ⭘ ⭘ ⭘

*Wenn „Eher selten“ oder „Nein“, über welche Probleme können Sie mit Ihrem Arzt/Ärztin nicht sprechen ? (Mehrfachantworten möglich)*

1. Psychologische Probleme (z.B. Ängste, Depressionen) 
2. Partnerschaftsprobleme 
3. Sexualität 
4. Körperliche Beschwerden (z.B. Schmerzen) 
5. Sonstige__________________________________________

15. *Wird während Ihrer Behandlung eine psychologische Betreuung angeboten?*

Ja 

Nein 

Zum Abschluss bitten wir Sie noch um einige Angaben zu Ihrer Person

16. *Ihr Alter:* _________

17. *Ihr Geschlecht:* weiblich 

männlich 

18. *Familienstand:*

**a) Verheiratet **

b) In einer festen Partnerschaft lebend 

1. c) Single 

d) Sonstiges________________________________________

19. *Haben Sie leibliche Kinder?*

Ja 

**Nein **

20. *Schulabschluss:*

a) Keinen 

b) Hauptschulabschluss 

c) Realschulabschluss 

d) Abitur 

e) Fachhoch/Hochschulabschluss 

21. *Lage und Größe Ihres Wohnorts:*

**a) Ländlich strukturiert **

**b) Kleinstadt **

**c) Mittelstadt **

**d) Großstadt **

22. *In welchem Bundesland bzw. Staat leben Sie?*

___________________________________

**Zusatzfrage während der Pilotphase**

23) *Hatten Sie Probleme beim Ausfüllen des Fragebogens?*

____________________________________
