# Supplementary material for: Information Needs and Visitors' Experience of an Internet Expert Forum on Infertility
Source: J Med Internet Res. 2005 Jun 30;7(2):e20. doi: 10.2196/jmir.7.2.e20 (PMC1550645; doi:10.2196/jmir.7.2.e20)
Supplement: Supplementary file 3 [file jmir_v7i2e20_app3.doc]

**Questionnaire**

Dear visitor to the expert forum,

**The Department of General Practice / Family Medicine and the Department of Obstetrics and Gynaecology / Study Group of Reproductive Medicine, University of Goettingen, are performing a study about the problems and the information needs of women and men with an unfulfilled desire for a child.**

We kindly ask you to answer the following questionnaire. Your answers will be kept in strict confidence. Regardless of whether you fill out the questionnaire or not, you will receive an answer. But, please keep in mind that the results of this investigation will benefit both you and others.

**If you would like to know more about us or if you would like to communicate with us, you can find more information about those responsible for it at** <http://www.allgemeinmedizin.med.uni-goettingen.de/> **or**

<http://wwwuser.gwdg.de/~ukfh/>

Many thanks for your interest!

[Filter question]

*Have you filled out this questionnaire before?*

Yes ⇒ [automatically back to the forum]

No ⇒ [directly to the next question]

I have no unfulfilled

desire for a child ⇒ [automatically back to the forum]

*Would you like to participate in the study and fill out the questionnaire?*

Yes ⇒ [directly to the questionnaire

No ⇒ [automatically back to the forum]

# Questionnaire:

1. *Who directed you to this questionnaire?*

1. Doctor 
2. Nurse/nursing auxiliary 
3. Friends/relatives/husband 
4. Journals/ television 
5. Found by chance 
6. Systematic search 
7. Others:________________________________________________

2. *Why did you visit the expert forum?*

(multiple answers possible)

**a) Searching for information, because my whish for
 a child has not been fulfilled **

b) I have questions about the causes of childlessness 

c) I have questions about different treatments 

d) I have questions about my current treatment 

e) I have questions about the results of a medical examination 

f) Others:__________________________________________________

3. *Is this the first time you have asked a question in the expert forum?*

Yes  [directly to question 8]

No  [directly to question 4]

# 4. *Did the expert forum satisfy your need for information?*

a) Yes 

b) No 

c) Partly 

Criticism/positive comments/wishes regarding the expert forum:

__________________________________________

5. *Have you discussed the answers from the experts with your doctor?*

**Yes **

**No **

6. *On reading the experts’ answers, did you then seek the advice of another
doctor or a specialized clinic??*

**Yes **

**No **

7. *Did you start a therapy on the basis of an expert’s advice ?*

**Yes **

**No **

8. *For how long already have you been confronted by the problem of childlessness“?*

1. **about __months**
2. **about __years**

9. *Whom did you ask for advice first?*

a) This expert forum 

b) Family doctor 

**c) Gynaecologist **

**d) Urologist **

e) Friends/relatives 

f) Minister/ priest 

g) Psychotherapist 

h) Alternative medicine 

i) Others_____________________________________________

10. *Are you currently receiving medical treatment?*

Yes  *If Yes, for how long*: a) for __months

b) for __years

No  [directly to question16]

11. *Which treatment are you currently receiving? (*multiple answers possible*)*

a) Hormones 

b) Assisted reproduction (IVF, ICSI, IUI) 

c) Psychotherapy 

d) Alternative methods (acupuncture, homeopathy) 

e) Operation 

1. f) Consultation 

g) Others____________________________________________

12. *How good is the medical care offered by your doctor or therapist? (This refers to doctors you are consulting.)*

##### **Medical care**

Very good Good Quite bad Very bad

**a) Gynaecologist ⭘ ⭘ ⭘ ⭘
b) Family doctor ⭘ ⭘ ⭘ ⭘
c) Doctor in a fertility**

**clinic ⭘ ⭘ ⭘ ⭘
d) Psychotherapist ⭘ ⭘ ⭘ ⭘
e) Andrologist ⭘ ⭘ ⭘ ⭘
f) Alternative medicine ⭘ ⭘ ⭘ ⭘
g) Others___________ ⭘ ⭘ ⭘ ⭘**

13. *How would you rate of the quality of the information and advice given by your doctor or therapist? (This refers to doctors you are consulting.)*

**Information/ Education**

Very good Good Quite bad Very bad

**a) Gynaecologist ⭘ ⭘ ⭘ ⭘
b) Family doctor ⭘ ⭘ ⭘ ⭘
c) Doctor in a fertility**

**clinic ⭘ ⭘ ⭘ ⭘
d) Psychotherapist ⭘ ⭘ ⭘ ⭘
e) Andrologist ⭘ ⭘ ⭘ ⭘
f) Alternative medicine ⭘ ⭘ ⭘ ⭘
g) Others___________ ⭘ ⭘ ⭘ ⭘**

###### What do you expect of your doctor:______________________________________

14. *Can you openly discuss your problems during therapy with your doctor? (This refers to doctors you are consulting.)*

**Yes Mostly Infrequently No**

**a) Gynaecologist ⭘ ⭘ ⭘ ⭘
b) Family doctor ⭘ ⭘ ⭘ ⭘
c) Doctor in a fertility**

**clinic ⭘ ⭘ ⭘ ⭘
d) Psychotherapist ⭘ ⭘ ⭘ ⭘
e) Andrologist ⭘ ⭘ ⭘ ⭘
f) Alternative medicine ⭘ ⭘ ⭘ ⭘
g) Others___________ ⭘ ⭘ ⭘ ⭘**

*If “Infrequently” or “No” which are the problems you can not discuss with your doctor? (*multiple answers possible*)*

1. Psychological problems (fears, depression) 
2. Problems in the partnership 
3. Sexual problems 
4. Medical condition (pain) 
5. Others__________________________________________

15. *Has your doctor offered you a psychological support during your therapy?*

**Yes **

**No **

Finally we would like to ask you some personal questions:

16. *Your age:* _________

17. S*ex:* female 

male 

18. *Family status:*

**a) Married **

b) Stable partnership 

1. c) Single 

d) Others________________________________________

19. *Do you have children who are biologically yours?*

Yes 

**No **

**20. *Education:***

a) Left school without qualification 

b) GSE 

c) A-levels 

d) University degree 

21. *Domicile:*

**a) Rural **

**b) Small town **

**c) Middle-sized town **

**d) Large city **

**22. *In which federal state/county or country do you live?***

___________________________________

**Additional question during the pilot phase:**

23) *Did you have problems filling out this questionnaire?*

____________________________________
