# Supplementary material for: Information Needs and Visitors' Experience of an Internet Expert Forum on Infertility
Source: J Med Internet Res. 2005 Jun 30;7(2):e20. doi: 10.2196/jmir.7.2.e20 (PMC1550645; doi:10.2196/jmir.7.2.e20)
Supplement: Supplementary file 4 [file jmir_v7i2e20_app4.doc]

# Himmel et al. (JMIR): Quotes in German

*In dem Ovulationskalender hab ich die fruchtbaren Tage bestimmen lassen, aber was heißt das nun ? Wenn die z.B. von So bis Do sind, soll man dann ab So bis Do jeden Tag GV haben oder lieber nur jeden 2. Tag, oder wie ist das gemeint um möglichst schnell schwanger zu werden ? Sorry, aber da hab ich völlig Unterschiedliches gehört.* (P110; FB 382.txt)

*Unser KIWU-Arzt hat uns nun zwecks Embryonentransfer zur Mini-Tet geraten.(...) Zudem rät er uns, ein sog. Assisted Hatching durchführen zu lassen.(...) Was halten Sie von dieser Methode? Angeblich soll sie nochmals die Schwangerschaftsrate erhöhen? Ich bin unsicher und habe natürlich auch Angst, dass dann, falls es zur Einnistung käme, ein behindertes Kind die Konsequenz wäre. Arbeiten Sie in T. auch mit solchen Methoden?* (P24; 12.txt)

*Nach einer neuerlichen Hormonuntersuchung teilte mein FA mir nun mit, dass meine Progesteronwerte katastrophal wären. Normal seien ein Wert zwischen 1000-2800 (???) und ich hätte 47. Somit wäre eine SS ausgeschlossen. Leider wurden mir die Werte nicht erklärt und auch keine Therapie vorgeschlagen. Können Sie mich aufklären? Habe ich ggf. keinen Eisprung und sind meine Werte wirklich so katastrophal? Diesen FA suche ich natürlich nicht nochmals auf. Raten Sie mir ggf. zu einer Kiwu-Sprechstunde oder kann ich selbst etwas tun?* (P196; FB 565.txt)

*... ich habe doch ein Recht zu erfahren was da im OP abgelaufen ist, oder liege ich da falsch? (...) Ich habe lediglich eine Kopie des Befundes des Einsendematerials bekommen (von d. Abrasio). Bitte übersetzen Sie mir dieses fachchinesisch, den ich bin fix und fertig weil ich das Gefühl habe er hatte mir etwas verschwiegen... ,denn der Arzt sagte mir ich wäre kerngesund gewesen. (...) Dadurch dass er mir den OP-Bericht verweigert bin ich nur noch mehr verunsichert*. (P105; FB 377.txt)

*Kiwu-Behandlung: 1. ICSI Punktion 09'2000: 14 Eizellen, alle befruchtet, 3 eingefroren, 2 zurück gesetzt bekommen, NEGATIV [...] 5. ICSI Punktion 02'2002: 7 Eizellen, 5 befruchtet, keine eingefroren!, 3 zurück gesetzt bekommen, NEGATIV Könnte es vielleicht doch mit meiner Endometriose zusammenhängen????? Ist vielleicht eine Wechseljahrtherapie oder eine Downregulation von 3 Monaten zu empfehlen? Oder ist eine Blutuntersuchung HLA zu empfehlen?? HILFE!!, ich weiß einfach nicht weiter, bin am Boden zerstört und völlig hilflos.* (P478; FB 1248.txt)

*Mein Freund sagt immer: Wenn ich mir so sehr ein Kind wünsche dann klappt es sowieso nicht. Das nimmt mir total meine Hoffnung. Er kann mich gar nicht verstehen wie ich mich fühle. Ich denke den ganzen Tag nur daran an ein Baby zu bekommen.* (P67; FB 294.txt)
